# Prévalence et connaissances de l’hypertension artérielle chez les personnes âgées: étude transversale menée à Bobo-Dioulasso, Burkina Faso

**DOI:** 10.11604/pamj.2018.30.243.15997

**Published:** 2018-08-01

**Authors:** Somnoma Jean-Baptiste Tougouma, Hervé Hien, Adjongba Bruno Aweh, Aimé Arsène Yaméogo, Ziemlé Clément Méda, Yibar Kambiré, Georges Rosario Millogo, Georges Kinda, Samba Sidibé, Macaire Ouédraogo

**Affiliations:** 1 Institut Supérieur des Sciences de la Santé (INSSA), Université Nazi Boni de Bobo Dioulasso (UNB), Burkina Faso; 2 Centre Muraz, Institut de Recherche en Sciences de la Santé, Bobo-Dioulasso, Burkina Faso; 3 Centre Hospitalier Universitaire Sourô Sanou, Bobo-Dioulasso, Burkina Faso; 4 Unité de Formation et de Recherche en Sciences de la Santé, Université de Ouagadougou, Burkina Faso; 5 Centre Hospitalier Universitaire du Point G, Bamako, Mali

**Keywords:** Hypertension artérielle, personne âgée, Bobo-Dioulasso, Hypertension, elderly, Bobo-Dioulasso

## Abstract

Le but de ce travail était de déterminer la prévalence de l'HTA chez les personnes âgées ainsi que leurs connaissances sur cette maladie. Il s'est agi d'une étude descriptive transversale qui s'est déroulée dans la ville de Bobo-Dioulasso d'Octobre à Novembre 2015 sur les sites d'intervention de l'association d'aide aux personnes âgées « KAFOLI ». Les sujets inclus étaient les personnes âgées de 60 ans et plus, hypertendus ou non et qui désiraient participer à l'étude. Les sujets étaient considérés comme hypertendus lorsqu'ils avaient une pression artérielle systolique ≥ 140mmHg et/ou une pression artérielle ≥ 90mmHg ou lorsqu'ils avaient un traitement antihypertenseur. Les données sociodémographiques, cliniques, les facteurs de risque associés ont été recueillis. Les connaissances sur l'HTA recueillies portaient sur les connaissances générales sur l'HTA, les sources d'informations sur l'HTA. 88 sujets ont été inclus dans notre étude. Il y avait 56 femmes et 32 hommes soit un sex-ratio de 0,57. L'âge médian était de 71 ans (IIQ: 66-76). La prévalence de l'HTA était de 61,36% et était associée à la connaissance de l'HTA et à la consommation d'alcool. 68,18% des patients avaient des connaissances sur l'HTA. La majorité était suivie dans les Centres de Santé de premier niveau par des infirmiers (64,81%). Cette étude a mis en évidence une forte prévalence de l'hypertension artérielle chez les personnes âgées à Bobo-Dioulasso. Ces personnes étaient en majorité sensibilisées sur la maladie. Leur suivi était assuré dans la majorité des cas par un personnel infirmier.

## Abstract (en)

This study aims to determine the prevalence of arterial hypertension (AH) in the elderly people as well as their knowledge of this disease. We conducted a cross-sectional, descriptive study in the town of Bobo-Dioulasso from October to November 2015 at the intervention sites of the Association of Medical Assistance to elderly people “KAFOLI”. Patients aged 60 years and more, with or without hypertension, who wished to participate in the study were included. Subjects were considered to be hypertensive when they had systolic blood pressure ≥ 140 mmHg and/or diastolic blood pressure ≥90 mmHg or when they were under antihypertensive treatment. Socio-demographic and clinical data as well as the risk factors associated with the disease were collected. Knowledges on arterial hypertension were based on general knowledges about arterial hypertension as sources of information about it. A total of 88 subjects were included in this study. The study involved 56 women and 32 men (sex ratio 0.57). The average age of patients was 71 years (IQR:66-76). The prevalence of arterial hypertension was 61,36% and it was associated with knowledges about AH and with alcohol consumption; 68.18 % of patients had knowledge of AH. The majority of them were followed up in first-level health care nursing centres (64,81%). This study highlighted a high prevalence of hypertension in elderly people living in Bobo-Dioulasso. The majority of these persons were aware of this disease. In the majority of cases follow-up was ensured by nursing staff.

## Introduction

L'hypertension artérielle constitue un problème majeur de santé publique dans les pays en développement. Elle toucherait 10 à 15% de la population adulte en Afrique noire [1, 2], avec des taux plus élevés en milieu urbain [3, 4]. La prévalence de l'HTA augmente avec l'âge [5]. Au Burkina Faso, la prévalence de l'HTA dans la population de personnes âgées demeure insuffisamment renseignée. Nous avons mené une étude transversale en milieu urbain, à Bobo-Dioulasso (Burkina Faso), dans le but d'y estimer la prévalence de l'HTA dans la population de personnes âgées. Un travail effectué en parallèle sur le même échantillon a permis d'évaluer le niveau d'information et les connaissances de cette population concernant l'hypertension artérielle.

## Méthodes

**Type et période d'étude:** Il s'est agi d'une étude transversale descriptive réalisée dans la ville de Bobo-Dioulasso. Elle s'est déroulée sur une période allant du 17 Octobre au 14 Novembre 2015.

**Site de l'étude:** L'étude a été réalisée sur quatre sites (secteur 9, secteur 12, secteur 18, secteur 24) répartis dans la ville de Bobo-Dioulasso. Nous avons fait un choix raisonné sur les sites d'activité de l'association KAFOLI.

**Population d'étude:** Cette étude s'est inscrite dans le cadre d'un suivi régulier annuel des personnes âgées de la ville de Bobo-Dioulasso mené par l'Association KAFOLI. KAFOLI a été créée le 21 Mai 2014 et est basée à Bobo-Dioulasso. C'est une Association d'assistance sociale aux personnes souffrant dans la vieillesse. L'association apporte des soutiens multiformes aux personnes âgées. Elles bénéficient entre autre d'un soutien médical, matériel, informationnel. L'adhésion à KAFOLI est libre pour toute personne intéressée par l'accompagnement et l'assistance sociale des personnes du troisième âge.

**Critères d'inclusion:** Tous les sujets hypertendus connus ou non, âgés d'au moins 60 ans révolus et consentant ont été inclus dans l'étude.

**Outils de collecte et déroulement de l'étude:** Les données ont été recueillies à l'aide d'une fiche de collecte de données qui reprenait l'ensemble des variables sur l'HTA et les connaissances. Une semaine avant le début de l'étude, et durant toute la période de collecte, l'association « KAFOLI » a initié des séances d'informations par différents canaux (médias audio, crieurs publics » dans les secteurs concernés par l'activité. La collecte des données s'est déroulée sur une période d'un mois. Les données sociodémographiques, cliniques, les facteurs de risque cardio-vasculaires et les connaissances sur l'hypertension artérielle ont été recueillis lors d'un entretien individuel couplé à une mesure des constantes, associé à une revue documentaire. Après administration du questionnaire au sujet, le carnet de santé était consulté au besoin pour plus d'information sur le profil tensionnel, l'existence ou non de pathologies chroniques associées comme le diabète et les dyslipidémies. Chaque site de collecte de données avait une équipe qui était composée de deux stagiaires internés de huitième année de médecine et de deux infirmiers. Les patients devaient observer un repos de 15 minutes au moins à leur arrivée sur le site de collecte. Après ce temps de repos, on procédait à l'administration du questionnaire puis la mesure des constantes. La tension artérielle était prise en position assise aux deux bras avec un tensiomètre automatique de marque OMRON étalonné et validé. Si elle était élevée, on procédait à une seconde mesure après cinq minutes. Les pèse-personnes étaient tous étalonnés, avec des cadrans à aiguille de graduation d'un kilogramme. Les enquêteurs ont bénéficié d'une mise à niveau après une séance de travail avec les médecins investigateurs.

**Echantillonnage:** Nous avons réalisé un échantillonnage raisonné pour le choix des sites. Tous les quatre sites animés par l'association « KAFOLI » ont été pris en compte pour la collecte des données. Tous les patients qui se sont présentés sur ces sites pendant la période d'étude et qui répondaient aux critères d'inclusion ont été retenus.

**Variables de l'étude:** Les données sociodémographiques (âge, sexe, niveau d'instruction scolaire, profession), les données cliniques (anthropométriques, pression artérielle aux deux bras, fréquence cardiaque, structure de suivi de l'HTA, qualificatif du personnel soignant), les facteurs de risque associés (la sédentarité, l'HTA, le diabète, le tabagisme, la consommation d'alcool) ont été recueillis. Les connaissances sur l'HTA recueillies portaient sur les connaissances générales sur l'HTA (signes cliniques, complications), les sources d'informations sur l'HTA (infirmiers, médecins généralistes, cardiologues, entourage, médias audio-visuels), le traitement de l'HTA (médicaments traditionnels, médicaments de la rue)

**Définitions opérationnelles:** 1) HTA: étaient hypertendus tous les patients qui avaient une pression artérielle systolique (PAS) ≥ 140mmHg et/ou une pression artérielle (PAD) ≥ 90mmHg ou lorsqu'ils avaient un traitement antihypertenseur quelque soit la régularité du traitement. 2) La personne âgée était une personne dont l'âge était ≥ 60 ans selon l'OMS [6]. 3) L'indice de masse corporelle calculé par le rapport poids/taille [7]. 4) La consommation d'alcool: définition inspirée de l'enquête STEPS 2013 [8]. Elle correspondait à une consommation de l'alcool au cours des 30 derniers jours ayant précédé l'enquête. On distinguait:

**la consommation abusive d'alcool:** Correspond à une consommation d'au moins 5 verres standards, en une seule occasion et ceci au moins 3 fois dans le mois chez les hommes, ou d'au moins 4 verres standards d'alcool en une seule occasion au moins deux fois dans le mois chez les femmes. Une consommation abusive d'alcool correspond à la prise d'une quantité moyenne d'alcool pur supérieure ou égale à 60g par jour pour les hommes et supérieure ou égale à 40g pour les femmes.

**La consommation moyenne:** Correspond à la prise d'une quantité moyenne d'alcool pur comprise entre 40g et 59,9g par jour soit 4 à 6 verres standards pour les hommes et entre 20g et 39,9g soit 2 à 4 verres standards pour les femmes.

**la faible consommation d'alcool:** Correspond à la prise d'une quantité moyenne d'alcool pur inférieure à 40g par jour pour les hommes et inférieure à 20g pour les femmes.

**Remarque:** Un verre standard contient 10g d'Ethanol. Par exemple: 1) un verre ou une coupe de vin correspond à un verre standard d'alcool; 2) une bouteille de bière de 33cl correspond à un verre standard d'alcool; 3) un ballon (boule) d'alcool fort correspond à un verre standard d'alcool; 4) une calebassée de bière de mil (dolo) correspond à un verre standard d'alcool; 5) une calebassée de vin de palm ou de bandji correspond à un verre standard d'alcool.

**Contrôle des Biais:** Les enquêteurs ont bénéficié d'une formation d'une semaine avec un médecin cardiologue et un médecin de santé publique. Cette formation a permis de passer en revue les techniques et conditions de prise de la pression artérielle, la conduite de l'interrogatoire et la revue documentaire. Le carnet de santé de chaque patient devait être recherché et exploité afin de minimiser les biais de mémorisation fréquente dans cette population.

**Considérations éthiques:** Le consentement éclairé était requis. La participation à l'étude était gratuite. L'étude s'est déroulée dans le strict respect de la confidentialité pour chaque patient. L'étude n'a pas exposé les patients à des risques additionnels. Des dispositions ont été prises et lorsque l'état du patient le nécessitait, il était référé au centre hospitalier universitaire Souro Sanou (CHUSS) pour une prise en charge adéquate.

**Saisie des données et analyse statistiques:** Une analyse descriptive simple a été réalisée sur l'ensemble de la population de l'étude. Les résultats étaient exprimés en fréquence pour les variables qualitatives ou en moyennes ± écart-type pour les variables quantitatives. L'estimation de la prévalence de l'HTA et des résultats moyens sur la population a été faite par un ajustement des données par rapport au sexe, avec un niveau de confiance statistique de 5%. Le test du khi carré de Pearson et le test exact de Fischer ont été utilisé pour la comparaison des pourcentages. La signification statistique était atteinte lorsque p < 0,05.

## Résultats

### Caractéristiques sociodémographiques et cliniques

123 personnes ont été reçues dans les sites durant la période d'étude, 88 correspondaient aux critères d'inclusion soit 71,54% des cas. Notre population d'étude était majoritairement féminine avec 56 femmes et 32 hommes soit respectivement 63,64% et 36,36% et un sex-ratio de 0,57. L'âge médian de notre population d'étude était de 71 ans (IIQ: 66-76). Le secteur le plus représenté était le secteur 12 avec 43% des participants, le moins représenté était le secteur 9 avec 11% des participants. La majorité des patients étaient sans instruction scolaire 69 soit 78,41%. 14 patients (16%) de notre population d'étude avaient toujours une activité professionnelle principalement le commerce et l'agriculture. 74 patients (84%) étaient sans activité professionnelle dont 14 (16%) bénéficiaient d'une pension de retraite trimestrielle.

### Prévalence de l'HTA

La pression artérielle systolique médiane était de 140 mmHg (IIQ: 130-150) et 90 mmHg (IIQ: 80-90) pour la pression artérielle diastolique. Dans notre population d'étude 54 personnes étaient hypertendues soit une prévalence globale de l'hypertension artérielle de 61,36%. Cette prévalence était de 59,38% chez les hommes et 62,50% chez les femmes. Cette différence n'était pas significative (p = 0,16). L'HTA systolique était la plus fréquente rapportée chez 34 patients (38,64%). La classification de l'HTA selon les différents grades est rapportée dans le Tableau 1. L'ancienneté de l'HTA était précisée chez 54 patients. Elle était pour 19 (35,19%), 14 (25,93%), 21 (38,89%) patients respectivement de moins de 5ans, 5 à 9 ans et supérieur ou égale à 10 ans. Les autres facteurs de risque associés étaient le diabète (6 patients soit 6,8%); le tabac (17 patients soit 19,3%); l'alcool (13 soit 14,8%); la sédentarité (15 patients soit 17,04%). L'obésité était rapportée dans 23 cas (17,05%). Il existait une association statistiquement significative entre HTA et consommation d'alcool (p = 0,01). L'HTA était insuffisamment traitée et seulement 18,5% des patients atteignaient les objectifs tensionnels. Elle était aussi mal traitée avec 31% des patients encore sous Furosémide 40mg seul comme traitement antihypertenseur. 19 patients (36,54%) ont déclarés avoir déjà interrompu leur traitement antihypertenseur. Les causes d'interruption de traitement étaient le manque de moyens financiers chez 9 patients (47%) suivies de l'arrêt volontaire chez 6 patients (31,6%). Les Centres de Santé et de Promotion Sociale (CSPS) étaient les structures de prise en charge de première intention (57 patients soit 64,8%). Aussi, seulement 19 patients soit 21,6% des patients étaient suivis par un personnel médical (médecins généralistes et cardiologues).

**Tableau 1:** Répartition des personnes âgées reçues dans les sites selon le grade de l’HTA; Bobo-Dioulasso, octobre-novembre 2015

Grade de l’HTA	Effectif (n)	Pourcentage (%)
TA normale	26	29,55
Grade 1	14	15,91
Grade 2	8	9,09
Grade 3	6	6,82
HTA systolique	34	38,64

TA: tension artérielle

**Figure 1:**
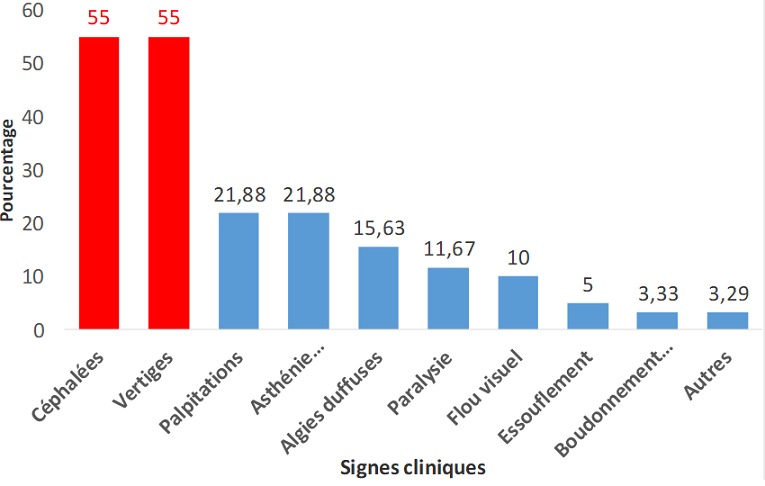
Connaissances des signes cliniques de l’HTA par les personnes âgées reçues dans les sites; Bobo-Dioulasso, Octobre-Novembre 2015

**Connaissances de l'HTA**: Dans notre étude 68,18 % des personnes âgées (n = 60) avaient déclaré avoir des connaissances sur l'HTA; tandis que 45,45% (n = 40) déclaraient connaitre leurs chiffres tensionnels habituels (Figure 1). Les signes les plus cités par les enquêtés étaient les céphalées et les vertiges dans 55% des cas respectivement suivi de l'asthénie physique et des palpitations avec 21,88% chacun. Les autres signes tels que la douleur abdominale, la lourdeur des membres, la prise de poids ont été cités dans 3,29% des cas. La prévalence de l'HTA était significativement plus élevée chez les personnes âgées qui avaient des connaissances sur l'HTA (p = 0,01).

**Sources d'informations sur l'HTA:** Les sources d'informations sur l'HTA des personnes âgées sont rapportées dans le Tableau 2. Les infirmiers représentaient la première source d'information des personnes âgées sur l'HTA avec 50% suivi de l'entourage à 23,33%. D'autres sources d'informations ont été également citées dans 11,67% des cas.

**Tableau 2:** Principales sources d’information sur l’HTA des personnes âgées reçues dans les sites; Bobo-Dioulasso, octobre-novembre 2015

Sources d’information	Effectif (n = 60)	Pourcentage (%)
Infirmiers	30	50
Entourage	14	23,33
Médecin généraliste	7	11,67
Autres	7	11,67
Cardiologue	2	3,33

**Régularité d'écoute des informations nationales sur les médias nationaux:** Dans notre étude, 92,05% soit 81 personnes âgées ont déclaré posséder un moyen audiovisuel mais 59,26% n'écoutaient pas régulièrement les informations nationales à travers les médias.

## Discussion

Notre étude présente quelques limites qu'il faut relever. Elle a en effet eu pour cadre, les sites d'activité d'une Association œuvrant au bénéfice des personnes âgées. A ce titre, il existe un biais de sélection en ce sens que ce sont les plus démunies qui ont souvent recours à ses services. En témoigne la faible participation du secteur 9 qualifié de zone résidentielle où les personnes âgées sont majoritairement des fonctionnaires retraités ayant suffisamment de moyens financiers pour une prise en charge personnalisée. Néanmoins nos résultats suscitent quelques commentaires discussions. L'HTA demeure un problème majeur de santé publique chez les personnes âgées. En témoigne notre prévalence élevée de 61,36%. Cette prévalence élevée est aussi rapportée dans plusieurs travaux africains. En effet, Hien au Burkina Faso [9] et Damorou au Togo [10] rapportaient respectivement des prévalences de 82 et 74,29% dans la population de personnes âgées. Cette forte prévalence, associée aux autres facteurs de risque cardiovasculaires augmente le risque cardio vasculaire global dans cette sous population.

Environ 2/3 de notre population d'étude avaient des connaissances sur l'HTA. Cette proportion est supérieure à celle rapportée par une autre étude en population menée au Burkina Faso en 2003 qui rapportait une proportion de 36% [5]. Cette augmentation serait due aux différentes campagnes de sensibilisation et à une meilleure formation des agents de santé pour une prise en charge efficiente de l'HTA. Seulement 18,5% des patients traités atteignaient les objectifs tensionnels. Ce constat est rapporté partout dans le monde et pas seulement en Afrique. En effet, seulement 25 à 30% des patients hypertendus dans le monde sont bien contrôlés par leur traitement antihypertenseur [11-14]. Dans toutes ces études, il ressortait que c'est surtout l'HTA systolique qui était le moins bien contrôlée, particulièrement chez le sujet âgé [15].

Il est donc nécessaire de motiver nos patients hypertendus par une information très précise sur les risques encourus à la suite d'une hypertension longtemps négligée. Pour cela, il est nécessaire d'avoir une bonne relation médecin-patient. Il faut entreprendre avec son patient une stratégie de prise en charge progressive de l'hypertension pour amener la pression artérielle vers une valeur cible qui est fixée dès le départ : inférieure à 140/90 mmHg pour la majorité des patients. Le choix des médicaments doit être raisonné en fonction des antécédents du patient, d'essais antérieurs, du risque possible d'effets secondaires, en fonction aussi du coût. Plus les médicaments sont nombreux et plus le coût s'élève. La personne âgée dans notre contexte de travail est très vulnérable. En effet, après le départ à la retraite, on observe un abandon médical de cette sous population avec des visites médicales de moins en moins fréquentes et une absence totale de système d'assurance maladie. Et pourtant, en période d'activité, ces patients bénéficiaient à travers leur employeur une assurance maladie leur permettant de faire face au coût des soins.

Nous avons noté aussi que 2/3 de nos patients étaient suivis dans les CSPS. Ces CSPS sont dirigés par des infirmiers et constituent le premier niveau de la pyramide de soins au Burkina Faso. La pénurie en médecins dans notre contexte oblige les patients à un suivi dans ces centres de premier niveau. Et pourtant, cette catégorie de personnel de premier niveau n'est pas souvent bien formée pour assurer cette prise en charge. Cela expliquerait le taux de 31% de patients sous monothérapie à base du furosémide 40mg. Il est donc nécessaire d'assurer une formation de qualité à cet échelon de la pyramide de soins d'une part et d'autre part, de créer des cadres de recyclage continu pour ces professionnels de santé dans le système de santé au Burkina Faso. Cela permettra sans doute un meilleur accompagnement de la santé de la personne âgée.

Malgré une forte proportion de possession de matériel audio-visuel, 60% environ de nos patients n'écoutaient pas régulièrement les informations sur le plan national. Ceci pourrait s'expliquer par un manque d'émission de sensibilisation et d'information en matière de santé dans nos médias publics. Pourtant l'IEC/CC (Information, Education et Communication pour le Changement de Comportement est un principe essentiel pour l'amélioration de la santé des populations dans notre contexte de pays à ressources limitées.

## Conclusion

L'HTA demeure un véritable problème de santé publique de nos jours. Sa prévalence est élevée chez la personne âgée. La majorité des personnes âgées connaissaient l'HTA en tant que pathologie. Leur suivi était assuré dans les CSPS par des personnels de santé non qualifiés pour la prise en charge de l'HTA.

### Etat des connaissances actuelle sur le sujet

- L'hypertension artérielle constitue un problème majeur de santé publique dans les pays en développement;
- La prévalence de l'HTA augmente avec l'âge;
- Au Burkina Faso, la prévalence de l'HTA dans la population de personnes âgées demeure insuffisamment renseignée.

### Contribution de notre étude à la connaissance

- La prévalence de l'HTA dans la population de personnes âgées est connue à Bobo-Dioulasso;
- Les connaissances sur l'HTA par les personnes âgées sont précisées;
- Les facteurs influençant une meilleure prise en charge de l'HTA des personnes âgées sont établis.

## Conflits d’intérêts

Les auteurs ne déclarent aucun conflit d'intérêts.
